# Need for a definitive trial of local versus general anaesthesia in chronic subdural haematoma; survey and literature review of ongoing trials

**DOI:** 10.1007/s00701-026-06982-5

**Published:** 2026-07-28

**Authors:** Daniel J. Stubbs, Conor S. Gillespie, Matthew L. Watson, Basil Nourallah, Caroline M. Phillips, George Gathercole, Jamie Brannigan, Keng Siang Lee, Orla Mantle, Vian Omar, Adele Mazzoleni, Githmi Palahepitiya Gamage, Alvaro Yanez Touzet, Munashe Veremu, Youssef Chedid, William H. Cook, Karanjit Loyal, Gideon Adegboyega, Oliver D. Mowforth, Edward Goacher, Apoorva Singh, Jonathan P. Coles, Alexis Joannides, Angelos Kolias, Judith Dinsmore, Iain Moppett, Michael Nathanson, Sally R. Wilson, Amit Deshmukh, Edoardo Viaroli, David K. Menon, Ellie Edlmann, Benjamin M. Davies, Peter J. Hutchinson, Improving Care In Elderly Neurosurgery Initiative (ICENI) Working Group

**Affiliations:** 1 Department of Perioperative, Acute, Critical Care, and Emergency Medicine (PACE) Section, University of Cambridge, Cambridge, CB2 0SZ UK; 2 Department of Neurosurgery, Addenbrooke’s Hospital, Cambridge, UK; 3 Department of Clinical Neurosciences, University of Cambridge, Cambridge, UK; 4 Department of Neurosurgery, King’s College Hospital, London, UK; 5 Department of Basic and Clinical Neurosciences, Maurice Wohl Clinical Neuroscience Institute, Institute of Psychiatry, Psychology and Neuroscience (IoPPN), King’s College London, London, UK; 6 Royal College of Surgeons of Ireland, Dublin, Republic of Ireland; 7 Department of Medicine, York and Scarborough Teaching Hospitals NHS Foundation Trust, York, UK; 8 Department of Neurosurgery, Hull Teaching Hospitals, Hull, UK; 9 Department of Neuroanaesthesia, St George’s Hospital, London, UK; 10 Academic Unit of Injury, Inflammation and Repair, University of Nottingham, Nottingham, UK; 11 Consultant Anaesthetist, Department of Anaesthesia, Nottingham University Hospitals NHS Trust, Nottingham, UK; 12 Department of Neuroanaesthesia, University College London Hospitals NHS Foundation Trust, London, UK; 13 Department of Neurosurgery, South West Neurosurgical Centre, Plymouth, UK

**Keywords:** CSDH, Anaesthesia, GA, LAS

## Abstract

**Background:**

Chronic subdural haematoma (cSDH) is a common neurosurgical condition, many patients have significant comorbidity or are living with frailty. Surgery is effective and can be performed under local anaesthesia (with or without sedation) (LAS) or general anaesthesia (GA). Optimal technique for both GA and LAS is poorly defined but similar questions have been explored in other surgical settings. We sought to clarify the breadth of evidence for anaesthetic technique in cSDH surgery, while drawing on relevant literature from other disciplines to understand how a definitive trial of this question could be performed.

**Materials and methods:**

We used a combination of review of trial registries, the Cochrane database, and a survey of anaesthetic and neurosurgical practitioners.

**Results:**

We identified a paucity of high-quality studies, especially randomised trials, exploring this question. The literature, and survey of anaesthetists and surgeons suggest that LAS may achieve equivalent outcomes, but significant confounding exists in current studies. Registered trials are single centre, and Cochrane reviews in other fields present issues of equipoise, study design, and outcome choice as key challenges in designing a definitive trial.

**Conclusions:**

There is significant interest in this topic as evidenced by published and emerging literature and views of anaesthetists and surgeons. No registered trial is multi-centre or draws on challenges identified in similar trials from other disciplines.

**Supplementary Information:**

The online version contains supplementary material available at https://doi.org/10.1007/s00701-026-06982-5.

## Introduction

A chronic subdural haematoma (cSDH) is a collection of altered blood beneath the dural membrane [47, 48]. It is associated with increasing age [55] and patients commonly have multiple co-morbidities or are living with frailty. Patients can present with a wide range of symptoms [8, 19]. cSDH may form with or without antecedent trauma [17]. Case numbers are projected to rise, with data from the United Kingdom (UK) and United States (US) suggesting the number of patients requiring operative management may rise by over 50% in the coming decades [17, 48, 51].

Treatment for symptomatic or large cSDH is primarily surgical [2, 24], although other methods have been investigated as alternative or adjuvant strategies [54, 55]. Although surgery is effective, perioperative morbidity including infection and recurrence can be significant [7, 41]. Various surgical techniques are used, including cSDH evacuation via burr hole, mini-craniotomy, or twist drill craniostomy [16]. Surgery can be performed under local anaesthesia with or without sedation (LAS) or general anaesthesia (GA), however evidence as to optimum technique is lacking [20, 49, 50]. This includes middle meningeal artery embolisation, with trial results yielding mixed results [21, 38, 44]. Previous systematic reviews on if LAS is feasible have sought to answer this question [39, 40] although data are scarce. Consequently, practice varies, often by country, with UK practice highly skewed towards the use of GA (93% of cases based on a 2016 national audit) [8].

In other disciplines, high-quality randomised controlled trials have sought to evaluate whether anaesthetic modality influences patient outcome [39]. Interpretation and conduct of such trials is difficult [43, 56] with challenges around patient selection, expertise, and equipoise. Although previous systematic reviews have revealed a paucity of high-quality randomised data examining anaesthetic modality for cSDH evacuation [40], a recent study has shown LAS use led to reduced complications and shorter hospital stay data [1]. Even in the context of equivalent longer-term outcomes, these short-term dividends may be meaningful for patients while offering productivity gains for pressured services [22]. Overall, this suggests a well-planned trial to answer this question is pressing. It remains unclear as to what extent current evidence answers this clinical question. In addition, the views of multi-disciplinary stakeholders in supporting and the feasibility of a trial is currently unknown. Patients with cSDH are often elderly and medically complex, and there is a need to examine if less invasive anaesthetic methods, in other surgical disciplines, could be explored and used.

Underpinned by a joint surgical and anaesthetic practitioner survey, and a literature review from other surgical disciplines, this article summarises current practice and explores why and how a comprehensive trial of anaesthetic modality for cSDH surgery might be conducted. The primary aim of this study was to identify if there is a requirement for a trial for GA vs LAS in cSDH, by examining current literature, expert consensus, and feasibility of method by comparing to other surgical conditions. The primary objective was to identify areas of consensus in modality from published literature, and carry out a survey of current practice.

## Methods

### Overview

Evidence is presented from two sources: a review of ongoing trials and studies from other surgical disciplines, and bespoke survey of surgical and anaesthetic practitioners.

### Survey of anaesthetic and surgical practitioners

Paired surveys to examine the perspectives of both surgeons and anaesthetists on the use of LAS in cSDH surgery were created. Questions examined the frequency with which a respondent was exposed to LAS use, relevant monitoring or pharmacological techniques employed, factors perceived as favouring general (GA) or local anaesthesia, and views on the need for a definitive trial. Exact questions posed to surgeons and anaesthetists are provided in supplemental methods. Surveys were distributed by email to members of the Society for British Neurological Surgeons (SBNS) and Neuroanaesthesia and Critical Care Society (NACCS) between December 2021 and January 2022 after review by the councils of both organisations. Surveys were distributed using the SurveyGalaxy software (www.surveygalaxy.com). As this was an anonymised survey of healthcare professionals, ethical review was not required [25]. Quantitative results are summarised in the text and supplemental results. Free-text responses to each question were thematically coded and mapped as to whether they represented advantages or disadvantages of conducting surgery under LAS.

### Identification of relevant ongoing trials

We searched the WHO International Clinical Trials Registry Platform (ICTRP) [53], clinicaltrials.gov [15], and ISRCTN database to identify ongoing or registered trials [32]. Trial databases were searched for ‘Chronic Subdural’ and all entries reviewed. Studies were included if they explored elements of anaesthetic technique of relevance to the conduct of cSDH surgery and were included if their status was listed as ‘recruiting’, ‘unknown’, or ‘completed with no results’.

### Learning from other surgical conditions

We searched the Cochrane database on 15th January 2025 to identify reviews of trials comparing local or regional anaesthetic techniques to general anaesthesia in other surgical conditions. We used the search terms ‘local versus general anaesthesia’ OR ‘regional versus general anaesthesia’ and included any review that compared these modalities in any surgical or procedural discipline. We included both local and regional (e.g. spinal or peripheral nerve block) anaesthesia as comparisons as it was felt that both comparators may share core issues of relevance to trial design in this setting.

## Results

### Ongoing studies on LAS v GA in cSDH

We identified 11 registered trials of relevance (Table 1). These were all single centre in nature and explored either LAS versus general anaesthesia (*n* = 3) or variants of LAS technique (*n* = 8). These included comparisons of the use of pre-operative melatonin, dexmedetomidine/ketamine versus dexmedetomidine/propofol, or adjunctive magnesium sulphate versus fentanyl alongside propofol for sedation. Many trials comparing LAS techniques featured dexmedetomidine as a comparator arm (*n* = 6). Postoperative inflammation is being assessed in one study [31].

**Table 1:** Registered clinical trials examining local anaesthetic (LAS) techniques in chronic subdural haematoma (cSDH) surgery

Year registered	Status	Setting	Study Design	Outcomes
2024 [31]	Pending	Single-centre Mansoura, Egypt	*Comparison:* general anaesthesia or awake surgery with dexmedetomidine sedation (1mcg/kg over 10 min followed by 0.2–0.7mcg/kg/hour) in cSDH patients undergoing craniotomy *Sample*: Target 62 *Inclusion*: Age ≥ 18 and ≤ 80 *Exclusion*: previous neurosurgery, other significant neurological disorder, severe medical comorbidities, allergies to study drugs, emergency surgery	• Postoperative inflammatory markers (CRP, IL-6, IL-8) • Stress hormones (cortisol, noradrenaline) pre- and postoperatively • Postoperative pain using VAS • Time to discharge from hospital • Postoperative complications • Time to first rescue analgesia • Postoperative mean arterial pressure • Postoperative heart rate
2024 [26]	Completed – no results listed	Single-centre Berlin, Germany	*Comparison:* local anaesthesia or general anaesthesia *Sample:* Target 50 *Inclusions:* Age ≥ 18, confirmed symptomatic cSDH, informed consent *Exclusions:* bilateral cSDH, preoperative delirium, allergy to local anaesthesia or adrenaline, psychiatric contraindication to awake surgery, > 24 h after admission, CSF shunt present	• Recruitment rate (consent rate and surgery rate) • Complication rate (discharge and 1 month) • Postoperative delirium • GCS at discharge and 1 month • Markwalder cSDH scale at discharge and 1 month • Barthel Index at discharge and 1 month • Mortality at 1 month • Length of stay • Discharge location • Health economic analysis • Reasons for non-participation
2023 [10]	Recruiting	Single-centre Beijing, China	*Comparison:* Scalp block + dexmedetomidine (2-4mcg/kg loading over 10 min then 0.5-1mcg/kg/hr to achieve RASS-3) *Sample:* Target 190 *Inclusions:* Age ≥ 18 and ≤ 80; haematoma thickness/shift > 1 cm *Exclusions:* GCS < 13, Sensory or motor aphasia, recurrent, severe comorbidity/other organ dysfunction, severe coagulopathy	• Intra-operative movement • Neurological function (discharge and 6 months) • Conversion to GA • Intraoperative awareness • Postoperative delirium • Recurrence at 6 months
2023 [30]	Complete – no results listed	Single centre Cairo, Egypt	*Comparison:* Scalp block with bupivacaine and lidocaine or scalp block with magnesium sulfate, bupivacaine and lidocaine in patients undergoing cSDH evacuation under general anaesthesia *Sample*: Target 38 *Inclusions:* ASA II-III, GCS > 13, age ≥ 45 *Exclusions:* patients needing postoperative ventilation, allergy to local anaesthetics, failed block, > 2 doses of rescue analgesia within the first postoperative hour, local infection, advanced cardiac pathology	• Time to first dose of postoperative analgesia • Perioperative heart rate • Perioperative systolic, diastolic and mean blood pressure • Postoperative pain using VAS
2022 [29]	Not Yet Recruiting	Number of centres not specified Varanasi, India	*Comparison:* Dexmedetomidine (1mcg/kg followed by infusion at 0.2–0.7mcg/kg/hr) or ketofol 1 mg/kg loading dose followed by infusion or ketofol 0.5 mg/kg/hour infusion in those undergoing burr hole evacuation of cSDH under monitored anaesthetic care *Sample*: Target 60 *Inclusions*: Age ≥ 25 and ≤ 60, ASA I – III *Exclusions*: Local infection at site of scalp block, uncontrolled hypertension, HR < 50, SBP < 90 mmHg, DBP < 60 mmHg	• Intraoperative haemodynamics • Surgeon satisfaction
2020 [14]	Completed – no results listed	Single centre, Zagazig, Egypt	*Comparison:* Dexmedetomidine-Ketamine *versus* Dexmedetomidine-Propofol titrated to achieve modified observer’s assessment of alertness and sedation score (OAA?S) 3 *Inclusions:* Age 50–80, BMI 25–30, ASA II/III, burr-hole evacuation *Exclusions:* Difficult airway (Mallampati III/IV), altered mental status, PTSD, baseline sedative/hypnotic medications, heart block, severe liver/respiratory/renal impairment	• Onset of sedation • Airway obstruction on a three-point scale • Intraoperative rescue analgesia (fentanyl) • Hypotension (MAP fall of > 20%), bradycardia (HR fall of > 20%), bradypnea (respiratory rate < 10), hypoxia (SpO2 < 95%) • Time to eye-opening • Neurological status using Markwalder’s Neurological Grading scale • Surgeon satisfaction (7 point LIKERT)
2019 [28]	Not Yet Recruiting	Number of centres not specified Kerala, India	*Comparison:* Scalp block + dexmedetomidine (0.2–0.5mcg/kg/hr) or propofol (25-50mcg/kg/min) *Sample:* Target 250 *Inclusions:* Age > 65, ASA II and III, cSDH for burr hole evacuation *Exclusions:* GCS < 15, repeat surgery, weakness of upper dominant limb, history of CVA, Parkinson’s disease, cognitive decline > 3 months, patient refusal, Mini Mental State Evaluation < 20 or Clinical Dementia Rating > 3	• Cognitive dysfunction (2 days, 3 months, 1 year post-op) • Functional outcome (2 days, 3 months, 1 year post-op) • Haemodynamic and ventilatory parameters
2018 [11]	Unknown	Single centre, Caen, France	*Comparison*: Xylocaine 1% for scalp block *versus* General Anaesthesia *Sample:* Estimated 60 *Inclusions:* Age ≥ 18, uni- or bilateral cSDH, French speaker *Exclusions:* Agitated/non-cooperative, other intracranial lesions, underlying neurological pathology, pregnant/lactating women	• Postoperative hospitalisation • Modified Rankin Score at 3 months • Surgical complications: ASDH, infection, hydrocephalus • Medical complications: Pneumonia, UTI, phlebitis, PE, other • Patient/surgeon satisfaction (Likert 5 point) • Cognitive impairment (MINI COG) at 6 months • Mortality at 6 months • Numeric pain rating scale at 2 days incl. opioid consumption • ICU admissions
2018 [13]	Completed – no results listed	Single centre, Cairo, Egypt	*Comparison:* Pre-operative oral melatonin versus placebo in those undergoing cSDH drainage under LA *Inclusions:* Age 50–65, ASA I – II, Unilateral, stable vital signs, GCS 14–15 *Exclusions:* GI symptoms, substance abuse, hepatic/renal insufficiency, antipsychotic/anticonvulsant/anti-parkinson medications; unable to tolerate propofol sedation	• Propofol consumption (in milligrams) • Blood pressure • Intraoperative patient movement • Time to recover from anaesthesia • Visual analogue score (VAS) pain • Time to first rescue analgesic
2018 [12]	Completed – no results listed	Single centre, Kasr El Aini, Egypt	*Comparison:* Non-inferiority comparison of magnesium sulphate versus fentanyl in comparison with propofol/lidocaine. Ramsay sedation score (RSS) of 3, with BIS sedation score of 60–80 *Inclusions:* Age > 50, ASA I-II, GCS 14–15, Unilateral cSDH *Exclusions:* Hypertension, Bradycardia, Ischaemic heart disease (in last 6 months), second/third degree heart block, Substance abuse, allergy, neuropsychiatric diseases, predicted difficult airway (Ganzouri score > 4)	• Propofol consumption • Patient movements • Intraoperative/postoperative haemodynamics • VAS for pain • Time to rescue analgesic • Adverse events • Surgeon satisfaction
2014 [27]	Completed – no results listed	Number of centres not specified Nihon, Japan	*Comparison:* dexmedetomidine versus propofol sedation for cSDH surgery under local anaesthesia *Sample:* Target 40 *Inclusions:* Age > 20, < 80 *Exclusions:* liver or renal dysfunction, arrhythmia	• Efficacy and side effects (details not specified)

In outcomes – bold = ‘primary’, non-bold = secondary. *ASDH* Acute subdural haematoma, *HR* Heart Rate, *ICU* Intensive Care Unit, *mcg* micrograms, *PE* Pulmonary Embolism, *UTI* Urinary Tract Infection, *VAS* Visual Analogue score

Most studies had restrictive inclusion criteria, excluding patients with significant baseline comorbidities, extremes of age, predicted airway difficulty, or low pre-operative GCS (Table 1). Outcomes were principally related to adequacy of technique (such as intra-operative movements or need for adjunctive propofol). Three studies stated they would examine surgeon satisfaction [11, 12, 14]; only one reported the intention of measuring patient satisfaction.

### Relevant studies in other conditions

Our search of the Cochrane database identified 12 systematic review records, although 3 (covering surgery for endovascular aortic aneurysm repair, vitrectomy, and cervical dilatation surgery) identified no relevant trials. Full details of identified Cochrane reviews are available in supplemental Table 1. Major results, grouped by surgical discipline are shown in Table 2. The 9 Cochrane reviews with data covered a variety of surgical procedures including hip fracture, carotid endarterectomy, general surgery, lower-limb revascularisation, gynaecological procedures, caesarean section, and breast cancer surgery. In total 163 randomised trials were included across these Cochrane reviews. Trials involved a multitude of comparisons dependent on the type of surgery including locoregional anaesthetic techniques and neuraxial blocks (e.g. spinal and epidural).

**Table 2:** Summary of Cochrane reviews examining locoregional anaesthesia and general anaesthesia

Surgical discipline	Number of studies	Study comparators	Outcomes examined	Findings
Breast Cancer	9–7 in meta-analysis	Paravertebral or other truncal anaesthetic *vs* general anaesthesia	Quality of recovery Pain Block complications Disease-free survival Chronic pain Postoperative quality of life	Regional anaesthesia may reduce pain and postoperative nausea and vomiting
Carotid Endarterectomy	16–14 in meta-analysis Dominated by the GALA trial of 4389 participants	Any locoregional technique (e.g. superficial/deep cervical plexus blocks)	Stroke Death Myocardial infarction Haemorrhage Cranial nerve injuries Blood pressure Patient/surgeon satisfaction	No difference except for a reduction in arterial shunting with local anaesthetic techniques
Caesarean section	22	Neuraxial	Death Wound/other infections Blood loss/change in Hb/Transfusion Neonatal outcomes Intraoperative pain Postoperative pain/PONV Maternal satisfaction	Reduced blood loss with neuraxial anaesthesia More women using GA said they would use the same technique again in subsequent LSCS
General Surgery	9 Cochrane reviews, 40 studies	Neuraxial techniques for any surgical procedure either as a sole comparator or adjunct to general anaesthesia	Mortality Pneumonia Myocardial Infarction ‘Serious Adverse Events’	Reduced 30-day mortality in those with neuraxial anaesthesia if intermediate/high cardiac risk. Reduced risk of pneumonia
Incomplete miscarriage	7 (although no high quality RCTs)	Paracervical local anaesthesia	Patient satisfaction Pain Mortality	Patient-centred outcomes NOT assessed despite being highly relevant
Hip Fracture	31–28 in meta-analysis	Neuraxial Peripheral nerve block (e.g. lumbar plexus block)	All-cause mortality Pneumonia Myocardial Infarction CVA Acute Confusion DVT Length of hospital stay Analgesia	Reduced DVT in the absence of intensive anticoagulation with regional anaesthesia. Unilateral spinal may reduce intraoperative hypotension
Lower-limb revascularisation	4	Neuraxial	Mortality CVA Myocardial Infarction Nerve dysfunction Amputation Hospital stay Postoperative cognitive dysfunction Pneumonia Recovery room complications Participant satisfaction Pain	Reduced rates of pneumonia with neuraxial anaesthesia
Paediatric herniorraphy	5	Regional anaesthesia (epidural, spinal, caudal)	Postoperative apnoeas/desaturation Neurodevelopment Failure rates Operator satisfaction Hospital/NICU/PICU stay	Reduced postoperative apnoeas if patients receiving sedation were excluded. Risk of failure with regional techniques
Radial Fracture	3	Nerve block Haematoma block IV Regional anaesthesia	Failure rates Anatomic alignment Clinical outcomes Resource use	Insufficient evidence

### Survey findings

We received 215 responses (*n* = 125 for anaesthesia, *n* = 89 for surgery). Denominator data is difficult to obtain but for surgery, and using published figures of approximately 1000 members, this would amount to a response rate of approximately 9% for surgeons. NACCS contains approximately 350 members, but as not all members are anaesthetists, an exact response rate is not possible to determine. General anaesthesia was the most common technique reported by 88% of surgeons and 66% of anaesthetists. Local anaesthesia was standard practice for only 6% of anaesthetic respondents. Both groups indicated that they believed LA could offer benefits in terms of length of stay (LOS) and postoperative complications.

**Fig. 1:**
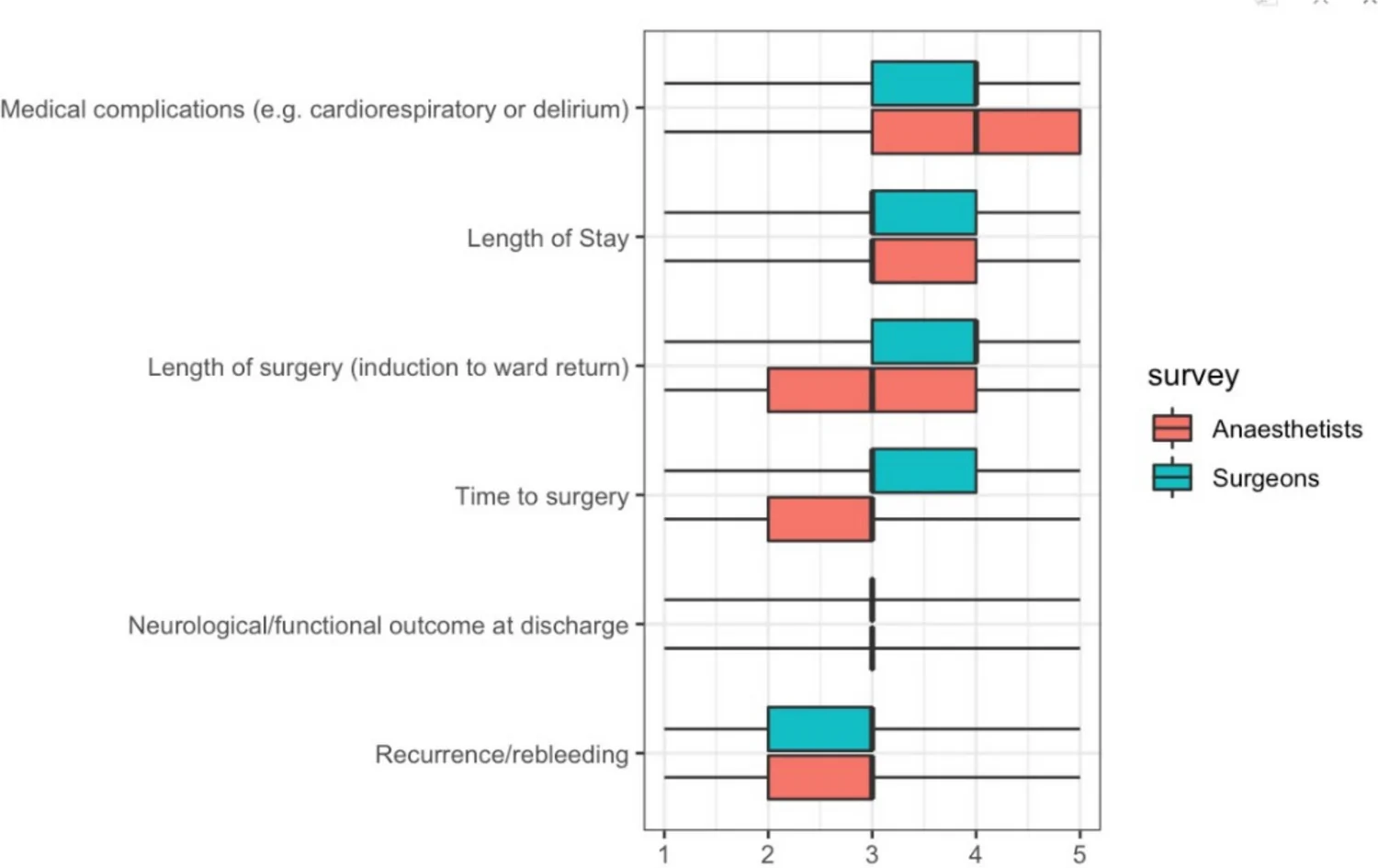
Perspectives of anaesthetists and surgeons on relation of postoperative outcomes to mode of anaesthesia (*1* = *strongly favours general anaesthesia, 3* = *equipoise, 5* = *strongly favours local anaesthesia).* Box plots indicate mean and interquartile range

**Fig. 2:**
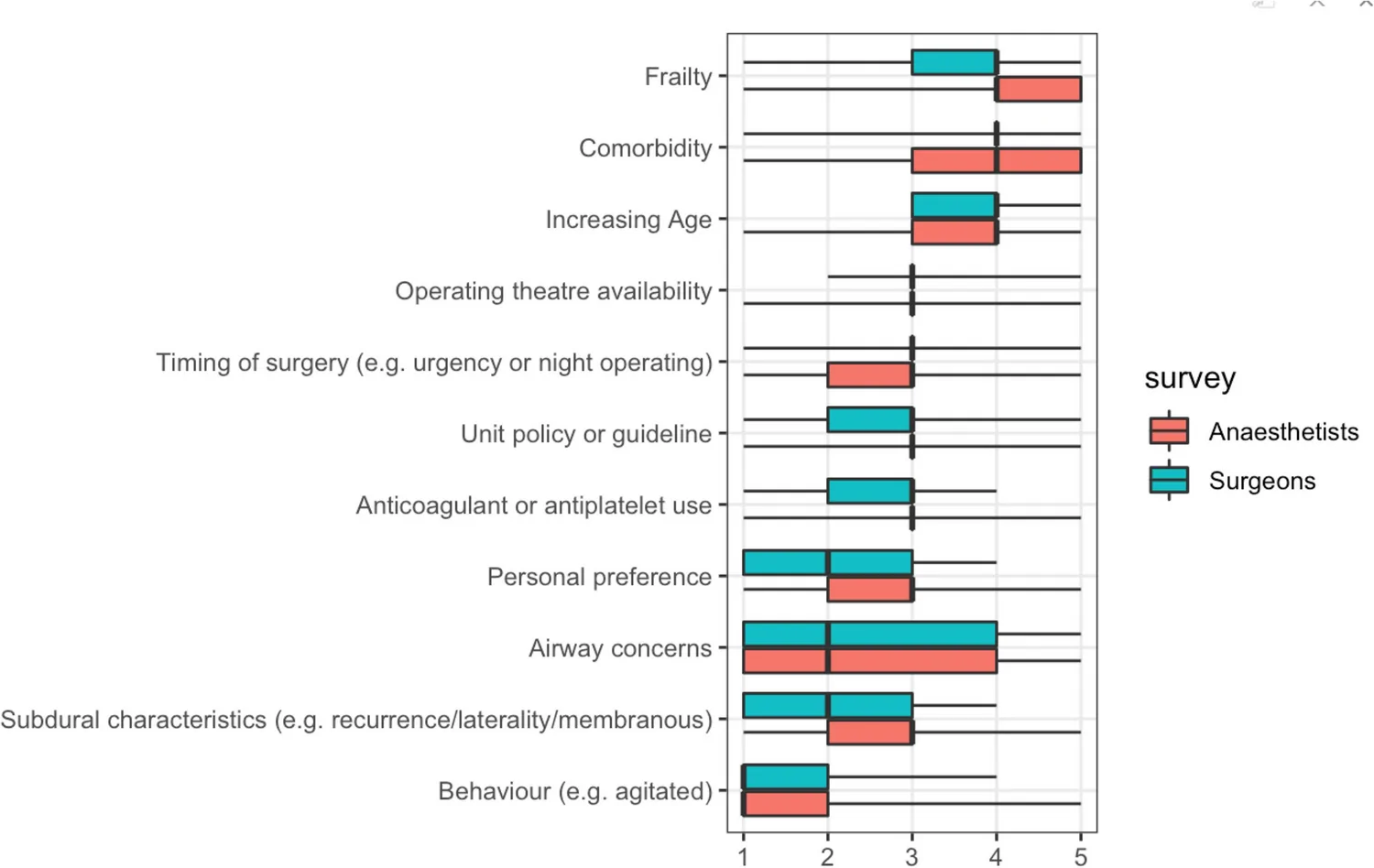
Perspectives of anaesthetists and surgeons on factors influencing choice of anaesthetic technique based on patient and operative characteristics. *(1* = *strongly favours general anaesthesia, 3* = *equipoise, 5* = *strongly favours local anaesthesia)*

Both surgeons and anaesthetists appeared to feel that comorbidities, frailty and increasing age favoured the use of LAS, while airway concerns and agitated behaviour strongly favoured general anaesthesia (Fig. 1 and 2). In general, views of anaesthetists and surgeons aligned, except on night-time operating (anaesthetists trend towards favouring GA in such a situation) and unit policies/antithrombotic use (surgeons trend towards favouring GA) [7].

**Fig. 3:**
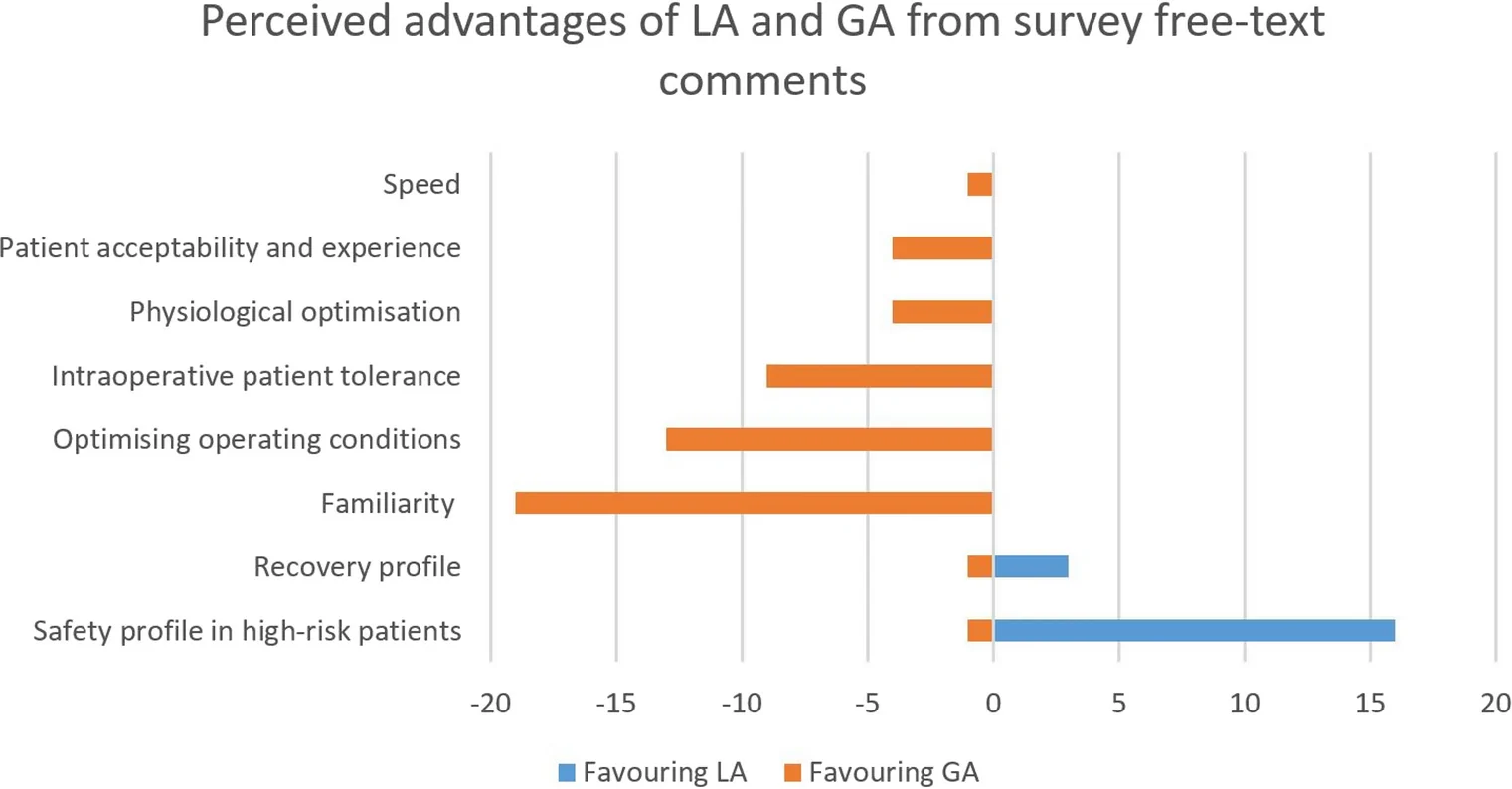
Major themes of anaesthetic and neurosurgical survey respondents on advantages of local anaesthesia (LAS) versus general anaesthesia (GA). Numbers refer to number of free-text comments received mapping to each theme

Thematically grouped free-text responses to questions were mapped as to whether they appeared to favour general or local anaesthesia (Fig. 3). These findings support the perception that LAS may bring benefits in terms of safety profile for high-risk individuals but that respondents indicate a lack of familiarity with LAS techniques, sub-optimal operative conditions, and tolerance.

Sedation with propofol alone was the commonest adjunctive LAS technique (23%). 53% of respondents chose intravenous maintenance for GA. 61% of respondents routinely used additional monitoring (26% arterial-line, 47% processed EEG). 61% of anaesthetic and 59% surgical respondents agreed that this question could benefit from further investigation with a randomised controlled trial. Importantly for any future trial a significant minority (~ 20%) of anaesthetic respondents indicated that they felt ‘not confident’ or ‘probably not confident’ in delivering LAS for cSDH surgery.

## Discussion

From our review of ongoing trials, there remains a paucity of high-quality evidence to inform choice of anaesthetic technique. From our survey, clinicians are also uncertain, with variation in practice and viewpoints, despite competency in both techniques. The survey data and analysis of registered trials, suggest that LAS may potentially reduce LOS, but this is confounded by its favoured use in those with better baseline neurology or smaller collections. The majority of anaesthetists and surgeons felt the choice of anaesthetic modality needed answering in a definitive RCT.

### Designing a trial- appropriate outcome selection

Evidence based medicine strives to improve patient outcomes and informative research must therefore ensure outcomes are patient centred. The challenge of applying this philosophy in perioperative research is well documented [33]. A scoping review of patient relevant outcome sets from multiple disciplines emphasises that complications, adverse events, and pain feature commonly [34]. The use of surrogates, whilst often objective and statistically powerful, can be difficult to interpret [33].

A possible measurable outcome in cSDH surgery could be LOS, which has not been a prominent outcome measure in cSDH research. LOS is associated with deconditioning and greater post-operative complications [3]. Work in other fields suggests patients view a short hospital stay as their second most important outcome in trial design [18]. From a service perspective, reduced LOS has health-economic and productivity benefits. This is of increasing significance, given the strained health service [9] and projected increased demand [48].

Recently, the GAP trial (Gabapentin versus Placebo as an adjunct to multimodal pain regiments) has used LOS (measured from the start of surgery to discharge) with secondary outcomes capturing clinical and patient quality of life metrics [4]. This indicates feasibility and may be replicated in cSDH.

An alternative approach, not examined in any of the identified cSDH literature, may be to evaluate ‘days-alive-and-at-home’ at either 30 or 90 days. This metric, validated in hip fracture and laparotomy [45], is viewed as patient-centric by simultaneously encompassing acute and subacute hospital stay, the impact of complications, mortality, and disability [33].

### Importance of patient experience

No studies in cSDH formally examined patient satisfaction of surgery conducted under local as opposed to general anaesthesia, although one study identified on a clinical trials registry did report on planning to examine this.

Patient satisfaction of LAS for cSDH evacuation was evaluated in one study using measurements of intraoperative and postoperative pain, satisfaction, hospital anxiety and depression (HAD) scores, and Impact of Event Scale – Revised (IESR) [43]. At 3–5 days postop and at 6 months, upwards of 75% of patients would agree to undergo the procedure again under LAS. With good intraoperative communication and reassurance, the study results suggest the procedure is relatively well tolerated.

Data from other conditions supports that overall locoregional anaesthesia is well tolerated, including in hand-surgery [36]. A prospective survey of patients undergoing carotid endarterectomy [6] demonstrated that older age and lower baseline cognition (assessed using the mini mental state examination – MMSE) had lower rates of pre-operative anxiety and lower requirements for preoperative information. This may be of relevance to cSDH surgery under LAS but formal examination of patient perspectives on acceptability are crucial. Our search of the Cochrane database (supplemental Table 1) demonstrated that both patient and surgeon satisfaction had been poorly examined across other similar trials in other disciplines.

### Currently used local anaesthetic techniques

Another area of uncertainty is the difference in reported LAS techniques and treatment regimens. This variability is shown in Table 3.

**Table 3:** Local anaesthetic techniques used in cSDH surgery

Study/Year	Local anaesthetic technique	Surgical technique	Other details	Failure rate (% convert to GA)
Ashry et al., 2022	Scalp block and midazolam (2–4 mg/hr) or propofol (1–3 mg/kg/hr)	2 × Burr hole		Not stated
Bishnoi et al. 2016 [5]	Dexmedotomidine (1 μg/kg over 10 min followed by continuous infusion 0.03 to 0.07 μg/kg/h) *vs* IV fentanyl 0.5 μg/kg and midazolam 0.03 mg/kg followed by 0.5 to 1.16 μg/kg/h fentanyl + 0.03—0.07 mg/kg/h midazolam + Lidocaine with adrenaline to incision site	Burr hole	Randomised (*n* = 52) Dexmedotomidine arm had fewer patient movement, better surgical satisfaction, and comparable patient satisfaction	0%
Hestin et al., 2022	Scalp block and remifentanil (0.5–1.5 ng/ml)	2 different methods used; burr hole craniostomy and craniotomy		7%
HM Wong et al., 2022	Scalp block and fentanyl (0.5–0.75 mg/kg)	Burr hole		0%
Mahmood et al. 2017	1–2 mg Midazolam then 2% Lidocaine without adrenaline	Mini craniotomy		Not stated
Raja et al. 2020	Dexmedotomidine 1ug loading followed by 0.3 µg/kg/min + fentanyl 1 µg/kg *vs* fentanyl 1 µg/kg + midazolam 0.03 mg/kg	Burr hole	Randomised *n* = *56*. Lower HR and blood pressure with dexmedotomidine. Higher PaO2 in recovery	Not stated
Srivastava et al. 2018 [46]	dexmedetomidine 1 μg kg^−1^ over 10 min as a loading dose, followed by 0.2–0.7 μg kg^−1^ h^−1.^ *Vs* propofol 1 mg kg^−1^ over 10 min as a loading dose, followed by 1–3 mg kg^−1^ h^−1^ alongside scalp block	Burr hole	Randomised. *N* = *62* One patient developed airway obstruction, two required vasopressors. Improved surgeon satisfaction with dexmedotomidine	0%
Surve et al., 2017	Local infiltration and dexmedetomidine (1mcg/kg loading over 10 min followed by 0.5mcg/kr/hr) and fentanyl bolus 1mck/kg) pre skin infiltration	Burr hole		8%
Taras et al., 2024	Local infiltration (2% Lidocaine) and diazepam (no dose details)	Burr hole		Not stated
Tuncer et al	Fentanyl/Propofol/Midazolam/Dexmedetomidine (no details)	Unilateral double burr hole		
Wang et al. 2016	Dexmedotomidine at 0.5 μg·kg^−1^ for 10 min *vs* Dexmedotomidine at 1.0 μg·kg^−1^ for 10 min *vs* Sufentanil 0.3 μg·kg^−1^ for 10 min	Burr hole	Randomised. *N* = 215 across three arms. Greater rates of rescue midazolam in low dose Dex. And Sufentanil groups. Better patient and surgeon satisfaction at high dose of Dex	0%
Zhuang et al., 2022	Local infiltration	Burr hole		0%

Four studies compared different LAS techniques [37, 42, 50]. Generally, Dexmedetomidine appears to be the most commonly utilised, but at different dosage protocols. Dexmedetomidine was assessed in all studies [52], and is deemed superior to propofol [35] and fentanyl with midazolam [12, 14], but optimal dosage remains to be elucidated. Given the wide variation in practice, a pragmatic design choice for a trial would be to leave technique to the individual anaesthetist. Although more reflective of real-world practice, high quality process measurements or clear definitions would need to be taken to ensure group separation – perhaps using processed EEG metrics and defining a general anaesthetic as one where advanced airway management is required (such as a supraglottic airway device or endotracheal tube).

### Challenges of conducting a randomised trial on anaesthetic modality

The 9 Cochrane reviews we identified (supplemental Table 1) demonstrated that similar questions of optimal anaesthetic technique, and the alternatives to general anaesthesia, have been explored across a variety of surgical disciplines.

Beyond methodological issues of randomisation quality and assessor blinding, heterogeneity of both anaesthetic and surgical technique, as well as a lack of equipoise in putting ‘high-risk’ patients forward for randomisation were apparent in the GALA study of GA versus LAS in carotid endarterectomy [23]. This might be especially relevant to cSDH given the potential for shared comorbidities between the cohorts and operative setup limiting access to the head/airway. A possible solution would be to control for this in trial inclusion criteria, identifying which modalities the patient may be eligible for on pre-operative assessment, before randomisation.

Many trials of locoregional versus general anaesthesia have permitted the co-use of sedative techniques as supplements. Being as many sedative agents can either be used as primary or adjunctive agents in general anaesthesia this creates the potential for contamination between comparison groups. Indeed, the RAGA trial in hip fracture surgery specifically examined spinal with no sedation as a comparator to general anaesthesia for this reason [37]. Future trials could include comparisons between GA, LAS without sedation, and LAS with titrated sedation, to avoid this contamination. This could incorporate main outcomes plus failure and conversion rates.

### Limitations

This study has several limitations. First, the review of ongoing trials is static, and trial results may be published which may help further define the evidence base. Second, the survey may be subject to response bias, with responders more likely to favour equipoise/interest in delivering LAS in cSDH. The review carried out was also not systematic in methodology or approach. It is therefore possible that some studies may be missed from our evidence synthesis. Nonetheless, the ongoing trials identified, and studies in other conditions indicate substantial heterogeneity with regards to patient selection, anaesthetic modality, and outcome measures. A clear consensus amongst clinicians is required for any definitive trial to be successful and avoid confounding.

## Conclusions

Definitive evidence for a specific anaesthetic modality in cSDH surgery is lacking. There is a suggestion that LAS may be noninferior to GA, with numerous trials ongoing. Using a survey of relevant practitioners, and a review of emerging and published literature demonstrates the need for a well planned, consensus based randomised trial in this field.

## Supplementary Information

Below is the link to the electronic supplementary material.

ESM 1 Supplementary Material 1 (DOCX 6.60 MB)

## Data Availability

All data are available (on reasonable request) by contacting the study corresponding authors.

## Abbreviations

CSDH: Chronic subdural haematoma
LAS: Local anaesthetic with or without sedation
GA: General anaesthetic
RCT: Randomised-controlled trial
NRT: Non-randomised trial
NOS: Newcastle Ottawa Scale
ICENI: Improving Care in Elderly Neurosurgery Initiative
