# Supplementary material for: Need for a definitive trial of local versus general anaesthesia in chronic subdural haematoma; survey and literature review of ongoing trials
Source: Acta Neurochir (Wien). 2026 Jul 28;168(1):246. doi: 10.1007/s00701-026-06982-5 (PMC13696589; doi:10.1007/s00701-026-06982-5)
Supplement: Supplementary file 1 [file 701_2026_6982_MOESM1_ESM.docx]

Need for a definitive trial of local versus general anaesthesia in chronic subdural haematoma; systematic review, survey, and scoping review.- supplementary material

**Authors and affiliations:**

Daniel J. Stubbs^1^, Conor S. Gillespie^2,3^, Matthew L. Watson^1^, Basil Nourallah^1^, Caroline M Phillips^1^, George Gathercole^1^, Jamie Brannigan^2,3^, Keng Siang Lee^4^, Orla Mantle^2,3^, Vian Omar^2,3^, Adele Mazzoleni^2,3^, Githmi Palahepitiya Gamage^5^, Alvaro Yanez Touzet^6^, Munashe Veremu^2,3^, Youssef Chedid^2,3^, William H Cook^2,3^, Karanjit Loyal^1^, Gideon Adegboyega^2,3^, Oliver D Mowforth^2,3^, Edward Goacher^7^, Apoorva Singh^1^, Jonathan P. Coles^1^, Alexis Joannides^2,3^, Angelos Kolias^2,3^, Judith Dinsmore^8^, Iain Moppett^9,10^, Michael Nathanson^10^, Sally R Wilson^11^, Amit Deshmukh^1^, Edoardo Viaroli^2,3^, David K. Menon^1^, Ellie Edlmann^12^, Benjamin M. Davies^2,3^, Peter J Hutchinson^2,3^, Improving Care In Elderly Neurosurgery Initiative (ICENI) Working Group

^1^Department of Medicine, Perioperative, Acute, Critical, and Emergency Care (PACE) Section, University of Cambridge, Cambridge, UK

^2^Department of Neurosurgery, Addenbrooke’s Hospital, Cambridge, UK

^3^Department of Clinical Neurosciences, University of Cambridge, Cambridge, UK

^4^Department of Neurosurgery, King's College Hospital, London, UK; Department of Basic and Clinical Neurosciences, Maurice Wohl Clinical Neuroscience Institute, Institute of Psychiatry, Psychology and Neuroscience (IoPPN), King's College London, London, UK

^5^Royal College of Surgeons of Ireland, Dublin, Republic of Ireland

^6^School of Medical Sciences, Faculty of Biology, Medicine and Health, University of Manchester, Manchester, UK

^7^Department of Neurosurgery, Hull Teaching Hospitals, Hull, United Kingdom

^8^Department of Neuroanaesthesia, St George's Hospital, London, UK

^9^Academic Unit of Injury, Inflammation and Repair, University of Nottingham, Nottingham, UK

^10^Consultant anaesthetist, Department of Anaesthesia, Nottingham University Hospitals NHS Trust, Nottingham, UK

^11^Department of Neuroanaesthesia, University College London Hospitals NHS Foundation Trust, London, UK

^12^Department of Neurosurgery, South West Neurosurgical Centre, Plymouth, UK

## Supplemental Methods

**Systematic review and meta-analysis of cSDH**

A systematic literature search was performed of Medline and Excerpta Medica Database (Embase), from inception to 2^nd^ January 2024. Search strategy for both databases can be found below. We reviewed the bibliographies of included articles to identify additional studies. Papers were limited to English language. The PRISMA checklist can be found in supplementary materials. The study was registered with PROSPERO (registration number: CRD42022374873). Articles addressing this review question were identified in parallel with studies addressing other research priorities [1] to support new guideline development [2]. Full details of article screening and risk of bias assessment are available in supplementary methods.

We pragmatically defined LAS as any anaesthetic technique involving the use of local anaesthetic including direct infiltration (field block) or scalp block. Patients could receive sedative medications but could not require advanced airway management (defined as need for an endotracheal tube or supraglottic airway device). General anaesthesia was defined as a technique involving the use of an equivalent advanced airway device with anaesthesia maintained by intravenous or inhalational agents.

We included studies examining the impact of anaesthetic technique on; postoperative complications, recurrence, mortality, hospital stay, and duration of anaesthesia and surgery. Postoperative complications were extracted from the included articles and included medical or surgical events occurring post evacuation during the same hospital admission. Definitions of recurrence were pragmatic based on individual studies but broadly considered ipsilateral CSDH significant enough to require re-operation. Postoperative mortality was defined as death within 3 months of CSDH evacuation. Duration of anaesthesia and surgery was the total time spent in theatre to undergo anaesthetic and surgery for CSDH evacuation (or as defined by study authors).

All statistical analysis was carried out using R Version 4.0.2 [3], using the meta package. Odds ratio (OR) meta-analyses using the Mantel–Haenszel method were computed for postoperative complication rate, recurrence rate and mortality rate. Mean difference meta-analyses were computed for length of stay (LOS) and duration of anaesthesia and surgery. Sensitivity analyses were performed excluding studies deemed at risk of bias; including studies scoring a ‘low risk of bias’ on the Cochrane risk of bias tool-2 or scoring seven to nine on the Newcastle Ottawa Scale (NOS) [4, 5]. Subsequent sub-group analyses were then performed between RCTs and NRTs to identify any effect that may be only due to observational studies. Heterogeneity was measured by the I^2^ test [6]. Funnel plots were used to assess publication bias, but were not generated if less than three studies were included [7].

**Search strategies**

A comprehensive literature search was performed on 1st May 2022 of 2 databases: Medline, and Excerpta Medica Database (Embase), from inception. The exact search strategy for all databases and their results can be found below.

MEDLINE 28^th^ April 2022

Embase 28^th^ April 2022

We reviewed the reference lists and bibliographies of included articles to attempt to identify additional studies. Papers were limited to English Language due to the difficulty in translation.

Study screening and selection

All identified citations were transferred to the online platform Rayyan [8], a repository to facilitate removal of duplicate records and independent screening of potential records. Duplicates were then removed. This article superset was used to inform all systematic reviews for guideline development [1]. Initially, each of these articles were screened by two blinded, independent reviewers. Articles matching inclusion criteria were then categorised dependent on their title, journal, and abstracts, into pre-defined categories corresponding to specific systematic review questions. These categories included:

1. Anticoagulant

2. Decision Making

3. Communication

4. Anaesthesia and Surgical Scheduling

5. Transfer and Pathway

6. Perioperative Care

7. Palliative Care

8. Postop and recovery

9. Natural history

10. Surgical technique

11. MMA Embolisation

We excluded studies that were conference abstracts, case reports, and mixed populations where it was not possible to delineate CSDH-specific results (e.g., a dataset containing both acute SDH and chronic SDH data). These abstracts were then assessed again using the population, intervention, comparison, outcome, and study design (PICOS) criteria defined in Supplementary Table 1 by two independent, blinded reviewers (MW, CG). These criteria were selected to identify studies pertinent to our primary aim. Any other articles that did not meet these criteria, but contained relevant information surrounding anaesthetic methodology for CSDH evacuation that could be used to address our secondary aim were separately identified.

**Supplementary Table 1. PICOS inclusion criteria with definitions for the primary aim.** PICO question derived from the output of a cross-disciplinary working group.

| **Review Question** | In adult patients undergoing surgical evacuation of a chronic subdural haematoma, does the use of local anaesthesia with or without sedation improve patient and system outcomes when compared to the use of general anaesthesia? |
| --- | --- |
| **Population** | Adult patients undergoing surgical evacuation of a chronic subdural haematoma |
| **Intervention** | The use of local anaesthetic with or without sedation for the operative procedure |
| **Comparator** | The use of general anaesthetic for the operative procedure |
| **Outcomes** | 1. Postoperative complication rate  2. Recurrence rate of subdural haematoma  3. Mortality rate postoperatively  4. Length of hospital stay  5. Duration of anaesthesia and surgery |
| **Study design** | Meta-analysis of defined outcomes using primary data from included studies |

If any disagreements occurred at either stage of screening, an attempt was made to resolve this between two review authors, and if discussion failed to lead to consensus, senior authors (BMD, DJS) were consulted for clarification. If any data was not present or available in the articles identified, corresponding authors were contacted via email to request the data.

Data extraction and synthesis

Data extraction was conducted independently and in duplicate by two authors using a standardised pre-piloted data collection proforma. Supplementary Files 4 and 5 are data collection proformas populated with relevant data for included studies.

**Supplementary Results**

Our initial search identified a superset of 6,049 primary research studies into CSDH 304 of these studies were of relevance to reviews within our ‘Anaesthesia and Surgical Scheduling’ theme. Following inclusion criteria, 140 articles were included in this study. Following free-text screening, 19 studies relating to primary and secondary aims were identified (**supplemental table 2)**. The PRISMA flowchart is shown

**PRISMA Diagram: Supplementary Figure 1**

| **Lead author of study** | **Type of publication** | **Year published** | **Risk of bias score** | **Country of the lead author** | **Number of cases** | **Number of LAS cases** | **Number of GA cases** | **Number of male cases** | **Mean age** | **Primary outcomes examined in study** | | | | |
| --- | --- | --- | --- | --- | --- | --- | --- | --- | --- | --- | --- | --- | --- | --- |
|  |  |  |  |  |  |  |  |  |  | **Post-operative complications** | **Recurrence rate of subdural haematoma** | **Mortality rate post-operatively** | **Length of hospital stay** | **Length of time in theatre** |
| Alnaami [9] | Retro and prospective cohort | 2021 | Low | Saudi Arabia | 88 | 41 | 47 | 71 | 64 | No data | Recorded and defined recurrence | No data | No data | No data |
| Ashry [10] | Retrospective cohort | 2022 | Medium | Egypt | 45 | 22 | 23 | 27 | 74 | Recorded postoperative complications | Do not define recurrence and no indication surgery was required | No data | Recorded mean length of hospital stay and standard deviation | Did not specify whether time recorded was including anaesthetic or not |
| Blaauw [11] | Retrospective cohort | 2020 | Low | Netherlands | 923 | 609 | 314 | 696 | 74 | Recorded postoperative complications | Recorded and defined recurrence | Recorded mortality data | Recorded mean length of hospital stay and standard deviation calculated | No data |
| Dakurah [12] | Retrospective cohort | 2006 | Low | Ghana | 96 | 12 | 84 | 90 | 47 | Recorded postoperative complications | Do not state which group had the recurrence | No data | No data | No data |
| Hestin [13] | Randomised control trial | 2022 | Low | France | 60 | 30 | 30 | 43 | 76 | Recorded postoperative complications | Recorded and defined recurrence | No data | Only reported median and IQR | Anaesthetic time not included |
| Hui Mei Wong [14] | Retrospective cohort | 2021 | Low | Malaysia | 257 | 130 | 127 | 201 | 68 | Recorded postoperative complications | Recorded and defined recurrence | Recorded mortality data | Recorded mean length of hospital stay and standard deviation calculated | No data |
| Mahmood [15] | Retrospective cohort | 2017 | Low | Pakistan | 35 | 16 | 19 | 25 | 69* | Recorded postoperative complications | Recorded and defined recurrence | Recorded mortality data | Recorded mean length of hospital stay and standard deviation calculated | Did not specify whether time recorded was including anaesthetic or not |
| Motiel-Langroudi [16] | Retrospective cohort | 2018 | Medium | America | 446 | 7 | 439 | 227 | 72 | No data | Data is based on re-operation (with other reasons such as acute SDH included) | No data | No data | No data |
| Oh [17] | Retrospective cohort | 2021 | Low | Korea | 293 | 87 | 206 | 208 | 75* | No data | Recorded and defined recurrence | No data | No data | No data |
| Phang [18] | Retrospective cohort | 2015 | Low | United Kingdom | 275 | 70 | 205 | 187 | 79* | No data | Recorded and defined recurrence | No data | No data | No data |
| Surve [19] | Randomised control trial | 2016 | Low | India | 69 | 35 | 34 | 65 | 58 | Recorded postoperative complications | Recorded and defined recurrence | Recorded mortality data | Recorded mean length of hospital stay and standard deviation | Recorded anaesthetic + surgery time |
| Tuncer [20] | Retrospective cohort | 2019 | Low | Turkey | 27 | 16 | 11 | 15 | 75 | No data | No data | No data | Recorded mean length of hospital stay and standard deviation | Recorded anaesthetic + surgery time |
| Zhuang [21] | Retrospective cohort | 2022 | Low | China | 105 | 51 | 54 | 91 | 65 | Recorded postoperative complications | Recorded and defined recurrence | No data | Recorded time from operation to discharge | Recorded anaesthetic + surgery time |
| Certo [22] | Retrospective cohort | 2019 | Medium | Italy | 45 | 30 | 15 | 28 | 76 | No data | Recorded and defined recurrence | No data | Recorded time from operation to discharge | No data |
| Taras [23] | Retrospective cohort | 2024 | Low | Ukraine | 67 | 23 | 44 | 51 | 60 | Recorded postoperative complications | Recorded and defined recurrence | No data | Recorded mean length of hospital stay and standard deviation | Did not specify whether time recorded was including anaesthetic or not |

**Supplemental Table 2: Table describing characteristics of published studies comparing local versus general anaesthesia on pre-specified outcomes. The lead author of the study is provided with the reference. The year the study was published, and the country of the lead author is provided. The number of cases, number of GA/LAS cases, number of male patients and average age for each study is described. Whether a study was included in the assessment of a primary outcome is displayed.** *Median age; this was provided if the study did not provide a mean. LAS; local anaesthetic with or without sedation. GA: general anaesthetic. *Green box: study included in initial meta-analysis and data recorded. Yellow box: study contained data related to primary outcome but was not used any further for reason cited. Red box: no data relating to the specific outcome in question*

| **Lead author of study** | **Postoperative complications examined in each study and their definition** | | | | |
| --- | --- | --- | --- | --- | --- |
|  | **Cardiac** | **Pulmonary** | **Neurological** | **Surgical** | **Other** |
| Ashry [10] | Hypertension  Acute coronary syndrome | - | Tension pneumocephalus  Seizures | Surgical site infection | Diabetic coma |
| Blaauw [11] | - | - | Delirium  Seizures | Rebleeding at wound site  Surgical site infection | Sepsis |
| Dakurah [12] | - | - | Tension pneumocephalus  Intracerebral haemorrhage  Cerebrospinal fluid leakage | - | - |
| Hestin [13] | Acute coronary syndrome | Dyspnoea | Delirium  Ischaemic stroke | Surgical site infection | Alcohol withdrawal  Urine infection |
| Hui Mei Wong [14] | Acute coronary syndrome | Pneumonia | Stroke  Intracerebral bleeds  Seizures | Surgical site infection | Thromboembolic events |
| Mahmood [15] | - | Pneumonia  Pleural effusion | Decreased GCS  Seizures | Rebleeding at wound site | Anaemia |
| Surve [19] | Bradycardia | Dyspnoea | Delirium  Decreased GCS  Tension pneumocephalus | - | Stridor  Throat pain  Diarrhoea |
| Zhuang [21] | - | Pneumonia  Dyspnoea | Delirium  Decreased GCS  Seizures  Meningoencephalitis | Poor wound healing  Drain insertion into cerebrum | Vomiting  Sore throat |
| Taras [23] | - | - | Tension Pneumocephalus | - | - |

**Supplemental table 3 Table identifying the different postoperative complications described in the initial studies that analysed postoperative complications. The complications described have been categorised by major organ system into cardiac, pulmonary, neurological, surgical, and other.**

| **Author/Study** | **Study design** | **Number of studies and patients** | **Outcomes and findings** | **Identified issues** |
| --- | --- | --- | --- | --- |
| General versus loco‐regional anesthesia for endovascular aortic aneurysm repair  Lee et al 2023  [24] | Types of studies: RCTs Population: patients undergoing endovascular abdominal or thoracic aortic aneurysm repair Intervention: loco-regional anaesthesia Comparison: general anaesthesia Primary outcomes: 30-day all-cause perioperative mortality, length of hospital stay, length of intensive care stay Secondary outcomes: incidence of endoleaks, requirement for re-intervention, incidence of myocardial infarction, quality of life (e.g. SF-36), incidence of respiratory complications, pulmonary embolism and deep vein thrombosis, length of procedure | No eligible studies identified | None | Absence of well-designed RCTs on this topic |
| Local versus general anaesthesia for carotid endarterectomy  Rerkasem et al, 2021  [25] | Types of studies: RCTs Population: people with symptomatic or asymptomatic carotid artery disease Intervention: local anaesthesia (any locoregional technique) Comparison: general anaesthesia Primary outcome: stroke of any kind within 30 days, and during long-term follow-up.  Secondary outcomes: death, stroke or death, myocardial infarction, local haemorrhage, cranial nerve injuries, blood pressure, shunted arteries, hospital stay, participant and surgeon satisfaction, and feasibility of performing operation under local anaesthesia | 16 studies (14 included in meta-analysis)  4839 participants | No significant difference in any stroke, death, ipsilateral stroke, stroke or death, myocardial infarction and other outcomes, except for a reduced rate of arterial shunting with local anaesthesia. | Dominated by GALA trial (3526 of 4839 participants), which did not have a prescriptive study protocol leading to a **wide variation** **in anaesthetic technique** (any general anaesthetic vs any locoregional technique) and surgical practice.  In GALA, **higher‐risk individuals**, who may have been felt to be better suited to a particular type of anaesthetic, **may not have been recruited**, as the overall stroke risks in the trial were very low  In some studies, **some randomised participants were removed from the analysis**, especially those who crossed over anaesthetic type  No studies able to blind participants or personnel to the mode of anaesthesia, introducing **high risk of performance bias**. 3 of 16 studies (including GALA) reported that outcomes were assessed by neurologists who were blinded to the type of anaesthesia used  **Variation in definition of some outcomes** (e.g. hypotension and hypertension) made these impossible to meta-analyse  Few trials assessed **surgeon or participant satisfaction** |
| Paravertebral anaesthesia with or without sedation versus general anaesthesia for women undergoing breast cancer surgery  Chhabra et al 2021  [26] | Types of studies: RCTs Population: adult women undergoing breast cancer surgery Intervention: paravertebral anaesthesia or other truncal regional anaesthesia, with or without sedation Comparison: general anaesthesia Primary outcomes: quality of recovery, postoperative pain at rest and on movement, mortality related to anaesthetic technique Secondary outcomes: adverse events due to paravertebral block, disease-free survival, chronic pain postoperatively, postoperative quality of life | 9 studies (7 included in meta-analysis)  614 participants | Paravertebral anaesthesia probably reduces 24-hour postoperative analgesic use, incidence of postoperative nausea and vomiting and pain at 2 hours, and may reduce pain at 24 hours at rest and pain on movement at 6 and 24 hours postoperatively compared to general anaesthesia. Reports of adverse events with paravertebral block were rare. | **Variation in surgical procedure** between and within studies, ranging from lumpectomy to modified radical mastectomy and reconstructive breast surgery  **Variation in blocks performed** (single- or multiple-level, with or without catheters)  **Variation in depth of sedation**, **and variation in sedative agents**. in one study some patients in the paravertebral group received no sedation and some received conscious sedation.  No assessment of quality of recovery **using a validated questionnaire**  **No assessment of disease-free survival, mortality, chronic pain or quality of life**, important metrics in breast cancer surgery  **High (but unavoidable) risk of performance bias** due to inability to randomise patients. In 4 studies there was **uncertainty about blinding of outcome assessors**. |
| Local versus general anaesthesia for adults undergoing pars plana vitrectomy surgery  Lincina et al 2016  [27] | Types of studies: RCTs or cluster-RCTs Population: adults undergoing pars plana vitrectomy Intervention: general anaesthesia Comparison: local anaesthesia (sub-tenon, peribulbar, retrobulbar, topical) Primary outcome: intraoperative adverse effects which may have a lasting effect on final visual outcome Secondary outcomes: postoperative adverse events, further application of local anaesthetic after start of surgery due to pain, patient satisfaction, degree of akinesia, surgical success | No eligible studies identified | None | Absence of well-designed RCTs on this topic |
| Anaesthesia for hip fracture surgery in adults  Guay et al 2016  [28] | Types of studies: RCTs Population: patients 16 or older undergoing emergency hip fracture repair surgery intervention: regional anaesthesia (neuraxial or peripheral nerve block such as lumbar plexus block), with or without sedation Comparison: general anaesthesia Primary outcomes: all-cause mortality at 30 days, pneumonia, myocardial infarction Secondary outcomes: cerebrovascular accident, acute confusional state, deep vein thrombosis, return to home, congestive cardiac failure, acute kidney injury, pulmonary embolism, unsatisfactory surgical results, number of patients transfused, length of hospital stay, length of surgery, intraoperative hypotension, urinary retention, incomplete or unsatisfactory analgesia | 31 studies (28 included in meta-analysis)  3231 participants | No differences except for reduced deep venous thrombosis in the absence of potent thromboprophylaxis with regional anaesthesia, and reduced intraoperative hypotension if unilateral or incremental spinal anaesthesia is used | **Suboptimal methodological rigour** in many trials, with respect to method used of randomization, concealment of allocation, **assessor blinding** and intention-to-treat analysis  **Definition of outcomes and time points for assessment** varied widely between studies (e.g. acute confusional state, intraoperative hypotension)  **General anaesthetic techniques unrepresentative of contemporary practice** (such as halothane maintenance) may have impacted results, and a correlation was demonstrated between the effect size for mortality and date of publication, suggesting that a lower mortality rate associated with regional anaesthesia was more pronounced in older trials |
| Regional (spinal, epidural, caudal) versus general anaesthesia in preterm infants undergoing inguinal herniorrhaphy in early infancy  Jones et al 2015  [29] | Types of studies: RCTs or quasi-RCTs Population: preterm infants born at less than 37 weeks’ gestation undergoing inguinal hernia repair before 60 weeks’ postmenstrual age Intervention: any form of regional anaesthesia (epidural, spinal or caudal) Comparison: general anaesthesia Primary outcomes: apnoea, desaturations, use of postoperative respiratory support, neurodevelopmental state at two-year follow-up Secondary outcomes: anaesthetic effectiveness with respect to operator satisfaction, anaesthetic failure, postoperative pain, duration of surgery, temperature on admission to recovery/NICU/PICU; duration of postoperative hospital stay, non-routine postoperative admission to PICU/NICU | 5 trials  152 participants | Significant reduction in postoperative apnoea in regional group once patients received preoperative sedation were excluded. Increased risk of anaesthetic agent failure or anaesthetic placement failure in the regional group. | **Poor quality of many included studies** – small sample size, poor randomisation, uncertain allocation concealment, incomplete outcome assessment blinding, inadequacy of intention-to-treat analysis  **Use of sedation in the regional group impacted results,** increasing the incidence of postoperative apnoea– only on the exclusion of patients receiving preoperative sedation was a significant difference between the groups detected  Pre-specified definition of apnoea in many studies inconsistent with accepted clinical definition |
| Neuraxial blockade for the prevention of postoperative mortality and major morbidity: an overview of Cochrane systematic reviews  Guay et al 2014  [30] | Types of studies: previous Cochrane systematic reviews Population: patients of any age undergoing surgery Intervention: neuraxial anaesthesia, or neuraxial anaesthesia plus general anaesthesia Comparison: general anaesthesia alone Outcomes: death, chest infection, myocardial infarction, serious adverse events | 9 Cochrane reviews  40 studies – half compared general versus neuraxial anaesthesia, the other half general plus neuraxial anaesthesia versus general anaesthesia alone | Neuraxial anaesthesia as a sole anaesthetic technique reduced 30-day mortality rate in patients undergoing procedures of intermediate to high cardiac risk, as well as reducing risk of perioperative pneumonia. | **Reporting of side effects and complications** related to anaesthetic techniques was incomplete  **Blinding was usually not used and was considered not feasible or realistic**, though some authors tried to insert a “sham” epidural catheter subcutaneously |
| Neuraxial anaesthesia for lower-limb revascularisation  Barbosa et al 2013  [31] | Types of studies: RCTs Population: adults aged 18 or older undergoing lower-limb revascularisation surgery Intervention: neuraxial anaesthesia Comparison: general anaesthesia  Primary outcomes: mortality, cerebral stroke, myocardial infarction, nerve dysfunction, rate of lower-limb amputation Secondary outcomes: duration of hospital stay, postoperative cognitive dysfunction, postoperative wound infection, pneumonia, complications in anaesthetic recovery room, participant satisfaction, postoperative pain score, transfusion requirement, urinary retention, claudication distance, pain at rest | 4 studies  696 participants | No difference observed in mortality rate, myocardial infarction and lower-limb amputation. Pneumonia was less common at neuraxial anaesthesia than general anaesthesia. Evidence was insufficient for other outcomes. | **Insufficient number of participants** to detect a meaningful difference in mortality  **Variations in anaesthetic technique incompletely reported** (e.g. spinal or epidural drugs, and level of blocks)  **Outdated anaesthetic technique** reported in older studies, limiting generalisability to modern practice  **Risk of selection bias** as most included studies did not sufficiently describe how random allocation and allocation concealment were achieved  **Most studies had unclear or inadequate blinding** of participants, study personnel and outcome assessors |
| Paracervical local anaesthesia for cervical dilatation and uterine intervention  Tangsiriwatthana et al 2013  [32] | Types of studies: randomised or controlled clinical studies Population: women undergoing cervical dilatation and uterine intervention Intervention: paracervical local anaesthesia Comparison: no treatment, placebo, other methods of regional anaesthesia, systemic sedation and analgesia, general anaesthesia Primary outcomes: | 26 studies  2790 participants | No studies found comparing general anaesthesia to paracervical local anaesthesia | Absence of well-designed RCTs on this topic |
| Anaesthesia for evacuation of incomplete miscarriage  Calvache et al 2012  [33] | Types of studies: RCTs or cluster-RCTs Population: patients with a diagnosis of incomplete miscarriage undergoing surgical evacuation Interventions: any anaesthetic technique given preoperatively or intraoperatively (general anaesthesia, sedation/analgesia, regional and paracervical local block) Comparison: any other anaesthetic technique Primary outcomes: patient satisfaction, pain during or after surgical evacuation, maternal mortality Secondary outcome: adverse events | 7 studies  800 participants | No studies found comparing general anaesthesia to locoregional anaesthetic techniques | Absence of well-designed RCTs on this topic  In other studies, **patient-centred outcomes such as patient satisfaction and quality of life were not assessed**, despite being highly relevant to mode of anaesthesia decisions in this procedure |
| Regional versus general anaesthesia for caesarean section  Afolabi et al 2012  [34] | Types of studies: RCTs or quasi-RCTs Population: women having elective or emergency caesarean section for any indication Intervention: regional anaesthesia, whether spinal, epidural or any combination of both Comparison: general anaesthesia  Primary outcomes:  Maternal: death, postoperative wound or other infections, difference between pre- and postoperative haematocrit or haemoglobin, blood loss > 500ml, mean blood loss, amount of blood transfused, number receiving postoperative blood transfusion Neonatal: death, mean umbilical arterial or venous pH, mean neurologic and adaptive score, mean Apgar at one and five minutes Secondary outcomes:  Maternal: incidence of intraoperative pain, maternal satisfaction, need for postoperative analgesia, incidence of postoperative nausea and vomiting, time to request postoperative analgesia, adverse events. Neonatal: time to sustained respiration, need for oxygen or intubation, Apgar score of 4, 6 or 8 or less at 1 and 5 minutes, mean Apgar score at 1 and 10 minutes | 29 studies met inclusion criteria but only 22 contributed data  1793 participants | No significant difference in major maternal or neonatal outcomes. Neuraxial anaesthesia associated with significantly lower estimated blood loss and drop in haematocrit. Significantly more women who had general anaesthesia stated that they would use the same technique again in a subsequent caesarean section. | Many of the included studies were **underpowered**  **Patient satisfaction was rarely assessed**, despite being an important and clinically relevant metric in caesarean section  Only one study analysed data in an **intention-to-treat** manner  Many studies **did not report method of randomisation or allocation concealment** |
| Anaesthesia for treating distal radial fracture in adults  Handoll et al 2002  [35] | Types of studies: RCTs or quasi-RCTs Population: patients who had completed skeletal growth receiving conservative or surgical treatment for a distal radius fracture Interventions: difference types of techniques of anaesthesia (nerve block, haematoma block, intravenous regional anaesthesia, sedation, general anaesthesia) Outcomes: failed/inadequate anaesthesia, anatomical restoration, adverse effects attributable to anaesthetic technique, clinical outcomes, functional outcomes, resource use | Of these, 3 trials involved general anaesthesia (versus haematoma block, sedation and haematoma block plus sedation)  At least 1200 participants | Insufficient robust evidence to establish relative effectiveness of different methods of anaesthesia in the treatment of distal radius fracture | **Inadequate reporting of trial methodology** in many studies, such as failure to ensure or confirm the concealment of allocation in all but 2 trials  Some trials **did not report highly relevant long-term outcomes** such as inadequate fracture reduction and functional outcomes  Studies involving general anaesthesia and sedation **did not report quantitative data on recovery time**  **Some studies exhibited confounders** such as imbalances between groups in fracture type and expertise of involved health professionals |

**Supplemental table 4: Cochrane reviews examining studies of locoregional versus general anaesthesia in other surgical disciplines.**

**Supplementary figure 2- Survey questions and responses to quantitative questions: Anaesthesia survey (Figures generated automatically via the Survey Galaxy software used to conduct surveys)**

**Please indicate your grade/experience**

**How frequently are you involved in the operative care of patients with chronic subdural**

**haematoma?**

**What percentage of your cases would you estimate you perform under local anaesthesia**

**(+/- sedation *without* the use of an artificial airway?**

*Free -text responses used to calculate mean of 19% with Standard deviation of 22%*

For responses given as e.g. <10% have taken midpoint between upper limit and next value of 10.

**What is your standard practice when anaesthetising patients with a chronic subdural**

**haematoma? ( *answer with your most commonly used technique* ). (Local anaesthesia infiltration/field block +/- sedation, Scalp block +/- sedation, General anaesthesia, No preference – situational judgement, Other)**

**Have you received any specific teaching or training on caring for patients with cSDH**

**having their operation under local anaesthesia +/- sedation?**

**Are you confident in caring for patients with a chronic subdural haematoma having their**

**surgery under local anaesthesia +/- sedation?**

When performing a case under MONITORED ANAESTHETIC CARE what

would be your preferred approach?

**I would use the following monitoring...**

**I would use the following sedative agents**

When performing a case under GENERAL anaesthesia what would your

preferred technique be?

**I would use the following monitoring...**

**Questions on ‘Influence of anaesthesia type on outcomes’ and patient/surgical factors influencing choice of anaesthetic technique are shown in figures 2 and 3 in the main paper.**

**Survey questions and responses to quantitative questions: Surgical survey**

**How frequently are you involved in the operative care of patients with chronic subdural**

**haematoma?**

**What percentage of your cases would you estimate you perform under local anaesthesia**

**(+/- sedation *without* the use of an artificial airway?**

*Free -text responses used to calculate mean of 13% with Standard deviation of 17%*

For responses given as e.g. <10% have taken midpoint between upper limit and next value of 10.

**Questions on ‘Influence of anaesthesia type on outcomes’ and patient/surgical factors influencing choice of anaesthetic technique are shown in figures 2 and 3 in the main paper.**

**References**

1.	Gillespie, C.S., et al., *How does research activity align with research need in chronic subdural haematoma: a gap analysis of systematic reviews with end-user selected knowledge gaps.* Acta Neurochir (Wien), 2023. **165**(7): p. 1975-1986.

2.	Stubbs, D.J., et al., *Protocol for the development of a multidisciplinary clinical practice guideline for the care of patients with chronic subdural haematoma.* Wellcome Open Res, 2023. **8**: p. 390.

3.	Gillespie, C.S., et al., *How should we treat long-standing overt ventriculomegaly in adults (LOVA)? A retrospective cohort study.* Neurosurg Rev, 2022. **45**(5): p. 3193-3200.

4.	Brannigan, J.F.M., et al., *Impact of antithrombotic agents on outcomes in patients requiring surgery for chronic subdural haematoma: a systematic review and meta-analysis.* Br J Neurosurg, 2024: p. 1-8.

5.	Gillespie, C.S., et al., *Genomic Alterations and the Incidence of Brain Metastases in Advanced and Metastatic NSCLC: A Systematic Review and Meta-Analysis.* J Thorac Oncol, 2023. **18**(12): p. 1703-1713.

6.	Doi, S.A., et al., *Advances in the meta-analysis of heterogeneous clinical trials I: The inverse variance heterogeneity model.* Contemp Clin Trials, 2015. **45**(Pt A): p. 130-8.

7.	Jonathan, A.C.S., et al., *Recommendations for examining and interpreting funnel plot asymmetry in meta-analyses of randomised controlled trials.* BMJ, 2011. **343**: p. d4002.

8.	Ouzzani, M., et al., *Rayyan-a web and mobile app for systematic reviews.* Syst Rev, 2016. **5**(1): p. 210.

9.	Alnaami, I., et al., *Surgery of chronic subdural hematoma under local anesthesia and its association with recurrence: A single tertiary care center experience.* Interdisciplinary Neurosurgery, 2021. **26**: p. 101293.

10.	Ashry, A., et al., *Local anesthesia versus general anesthesia for evacuation of chronic subdural hematoma in elderly patients above 70 years old.* Surg Neurol Int, 2022. **13**: p. 13.

11.	Blaauw, J., et al., *Neurosurgical and Perioperative Management of Chronic Subdural Hematoma.* Front Neurol, 2020. **11**: p. 550.

12.	Dakurah, T.K., et al., *Chronic subdural haematoma: review of 96 cases attending the Korle Bu Teaching Hospital, Accra.* West Afr J Med, 2005. **24**(4): p. 283-6.

13.	Hestin, R., et al., *Time to medical fitness for discharge following surgical evacuation of chronic subdural haematoma with general or regional anaesthesia: a prospective randomised trial.* Anaesthesia, 2022. **77**(5): p. 547-554.

14.	Wong, H.M., et al., *Chronic Subdural Hematoma Drainage Under Local Anesthesia with Sedation versus General Anesthesia and Its Outcome.* World Neurosurg, 2022. **157**: p. e276-e285.

15.	Mahmood, S.D., et al., *Mini-Craniotomy Under Local Anesthesia for Chronic Subdural Hematoma: An Effective Choice for Elderly Patients and for Patients in a Resource-Strained Environment.* World Neurosurg, 2017. **106**: p. 676-679.

16.	Motiei-Langroudi, R., et al., *Factors predicting reoperation of chronic subdural hematoma following primary surgical evacuation.* J Neurosurg, 2018. **129**(5): p. 1143-1150.

17.	Oh, H.J., et al., *Clinical Characteristics and Current Managements for Patients with Chronic Subdural Hematoma : A Retrospective Multicenter Pilot Study in the Republic of Korea.* J Korean Neurosurg Soc, 2022. **65**(2): p. 255-268.

18.	Phang, I., R. Sivakumaran, and M.C. Papadopoulos, *No association between seniority of surgeon and postoperative recurrence of chronic subdural haematoma.* Ann R Coll Surg Engl, 2015. **97**(8): p. 584-8.

19.	Surve, R.M., et al., *Use of Dexmedetomidine Along With Local Infiltration Versus General Anesthesia for Burr Hole and Evacuation of Chronic Subdural Hematoma (CSDH).* J Neurosurg Anesthesiol, 2017. **29**(3): p. 274-280.

20.	Tuncer, C. and Ö. Polat, *Opinions on Using Operating Room Effectively in Chronic Subdural Hematoma Surgery.* Duzce Medical Journal, 2019. **21**(2): p. 123-126.

21.	Zhuang, Z., et al., *Using Local Anesthesia for Burr Hole Surgery of Chronic Subdural Hematoma Reduces Postoperative Complications, Length of Stay, and Hospitalization Cost: A Retrospective Cohort Study From a Single Center.* Front Surg, 2022. **9**: p. 783885.

22.	Certo, F., et al., *Pros and cons of a minimally invasive percutaneous subdural drainage system for evacuation of chronic subdural hematoma under local anesthesia.* Clin Neurol Neurosurg, 2019. **187**: p. 105559.

23.	Havryliv, T., et al., *Single-Center Comparison of Chronic Subdural Hematoma Evacuation Outcomes Under Local Versus General Anesthesia.* World Neurosurg, 2024. **184**: p. e39-e44.

24.	Lee, S., et al., *General versus loco-regional anesthesia for endovascular aortic aneurysm repair.* Cochrane Database Syst Rev, 2023. **4**(4): p. Cd013182.

25.	Rerkasem, K. and P.M. Rothwell, *Local versus general anaesthesia for carotid endarterectomy.* Cochrane Database Syst Rev, 2008(4): p. Cd000126.

26.	Chhabra, A., et al., *Paravertebral anaesthesia with or without sedation versus general anaesthesia for women undergoing breast cancer surgery.* Cochrane Database Syst Rev, 2021. **2**(2): p. Cd012968.

27.	Licina, A., et al., *Local versus general anaesthesia for adults undergoing pars plana vitrectomy surgery.* Cochrane Database Syst Rev, 2016. **9**(9): p. Cd009936.

28.	Guay, J., et al., *Anaesthesia for hip fracture surgery in adults.* Cochrane Database Syst Rev, 2016. **2**(2): p. Cd000521.

29.	Jones, L.J., et al., *Regional (spinal, epidural, caudal) versus general anaesthesia in preterm infants undergoing inguinal herniorrhaphy in early infancy.* Cochrane Database Syst Rev, 2015. **2015**(6): p. Cd003669.

30.	Guay, J., et al., *Neuraxial blockade for the prevention of postoperative mortality and major morbidity: an overview of Cochrane systematic reviews.* Cochrane Database Syst Rev, 2014. **2014**(1): p. Cd010108.

31.	Barbosa, F.T., et al., *Neuraxial anaesthesia for lower-limb revascularization.* Cochrane Database Syst Rev, 2013. **2013**(7): p. Cd007083.

32.	Tangsiriwatthana, T., et al., *Paracervical local anaesthesia for cervical dilatation and uterine intervention.* Cochrane Database Syst Rev, 2013. **2013**(9): p. Cd005056.

33.	Calvache, J.A., et al., *Anaesthesia for evacuation of incomplete miscarriage.* Cochrane Database Syst Rev, 2012. **2012**(4): p. Cd008681.

34.	Afolabi, B.B. and F.E. Lesi, *Regional versus general anaesthesia for caesarean section.* Cochrane Database Syst Rev, 2012. **10**(10): p. Cd004350.

35.	Handoll, H.H., R. Madhok, and C. Dodds, *Anaesthesia for treating distal radial fracture in adults.* Cochrane Database Syst Rev, 2002. **2002**(3): p. Cd003320.
